# Draft Genome Sequence of *Geobacillus thermopakistaniensis* Strain MAS1

**DOI:** 10.1128/genomeA.00559-14

**Published:** 2014-06-05

**Authors:** Masood Ahmed Siddiqui, Naeem Rashid, Saravanaraj Ayyampalayam, William B. Whitman

**Affiliations:** a Department of Chemistry, Biotechnology Research Laboratory, University of Balochistan, Quetta, Pakistan; b School of Biological Sciences, University of the Punjab, Quaid-i-Azam Campus, Lahore, Pakistan; c Institute of Bioinformatics, University of Georgia, Athens, Georgia, USA; d Department of Microbiology, University of Georgia, Athens, Georgia, USA

## Abstract

*Geobacillus thermopakistaniensis* strain MAS1 was isolated from a hot spring located in the Northern Areas of Pakistan. The draft genome sequence was 3.5 Mb and identified a number of genes of potential industrial importance, including genes encoding glycoside hydrolases, pullulanase, amylopullulanase, glycosidase, and alcohol dehydrogenases.

## GENOME ANNOUNCEMENT

Thermostable enzymes isolated from thermophilic bacteria have been studied for the last two decades for many reasons, but especially for their potential biotechnological and industrial applications (1). Enzymes from species belonging to the genus *Geobacillus* have been characterized for industrial uses, including amylase, amylopullulanase, DNA polymerase, superoxide dismutase, xylanase, and lipase.

The thermophilic bacterium *Geobacillus thermopakistaniensis* strain MAS1 was isolated from a hot spring located in the Northern Areas of Pakistan. In order to identify genes of biotechnological interest, a draft genome assembly was constructed. The genome shotgun library was sequenced using the Illumina HiSeq 2500 platform with 150-bp paired-end reads. The draft genome was assembled using the a5 microbial genome assembly pipeline (2). The Rapid Annotations using Subsystems Technology (RAST) 4.0 server (3) and NCBI Prokaryotic Genome Annotation Pipeline (http://www.ncbi.nlm.nih.gov/genome/annotation_prok/) were used for genome sequence annotation. RNAmmer 1.2 and tRNA-scan-SE 1.21 were used to predict rRNAs and tRNAs, respectively (4, 5).

The genome sequencing generated 651,994 reads covering a total of 97.8 Mb or approximately 28-fold average genome coverage. There were a total of 121 contigs with a total length of 3,497,407 bp and with an *N*_50_ contig size of 62,025 bp. There were 117 contigs greater than 500 bp, and only 4 contigs were less than 500 bp but greater than 400 bp. The G+C content was 52.2%. The total numbers of genes and coding DNA sequences (CDSs) identified were 3,767 and 3,525, respectively. 16 rRNA genes, 94 tRNA genes, and 111 frameshifted genes were also identified.

The genome of strain MAS1 contains many *α*-glucan active enzyme-encoding genes, including those encoding six glycoside hydrolases (T260_04695, T260_10185, T260_10190, T260_10195, T260_11955, T260_12595), pullulanase (T260_01005), amylopullulanase (T260_04900), and glycosidase (T260_13695). It also contains five types of alcohol dehydrogenases (T260_01930, T260_06755, T260_07340, T260_09050, T260_12960) and lipases (T260_04645, T260_04700, T260_15915, T260_16665, T260_19075). According to the protein homology search analysis using the Basic Local Alignment Search Tool (BLAST) for five open reading frames annotated as glycoside hydrolases from *G. thermopakistaniensis* sp. MAS1, the MAS1 gene T260_11955 possesses homologs among almost all *Geobacillus* species, while homologs of the MAS1 genes T260_10185, T260_10190, T260_10195 are only present in *Geobacillus* sp. strain C56-T3. In addition, the MAS1 genes T260_04695 and T260_12595 are found in only *Geobacillus* sp. JF8. Some glycoside hydrolases from *Deinococcus geothermalis*, *Clostridium termitidis, Closterium saccharobutylicum*, *Thermobacillus composti*, *Bacillus cellulosilyticus*, *Paenibacillus dendritiformis*, and *Thermoanaerobacterium thermosaccharolyticum* also possessed high similarity to the *Geobacillus* enzymes. Thus, the distribution of these industrially important enzymes appears to depend upon strain and not species or genus characteristics. Furthermore, strain MAS1 possesses a unique combination of glycosyl hydrolases genes and is a good candidate for further analyses.

### Nucleotide sequence accession numbers.

The complete genome sequence of *Geobacillus thermopakistaniensis* strain MAS1 is now available at DDBJ/EMBL/GenBank database under accession no. AYSF00000000. The version described in this paper is version AYSF01000000.
